# Blistering Hot Topic: A Rare Case of Staphylococcal Scalded Skin Syndrome

**DOI:** 10.7759/cureus.90987

**Published:** 2025-08-25

**Authors:** Fariha Bhuiyan, Christopher T Gabbert, Nonna L Ebalo

**Affiliations:** 1 Sentara Halifax Regional Hospital Clinical Site, Edward Via College of Osteopathic Medicine, South Boston, USA; 2 Pediatric Medicine, South Boston Pediatrics, South Boston, USA

**Keywords:** exfoliative toxin a, painful skin lesions, pediatric derm, s aureus, staphylococcal scalded skin syndrome

## Abstract

Staphylococcal scalded skin syndrome (SSSS) results from dermatological infection with toxin-producing *Staphylococcus (S.) aureus*, consequently leading to exfoliative epidermal sequelae. This infection can be life-threatening, but generally resolves with an antibiotic regimen, and typically affects children under the age of two, with rare occurrence in children over the age of six. This case involves an eight-year-old unimmunized female, with no significant past medical history, who presented to an outpatient pediatric clinic with concerns of a painful rash that began on the face within 24 hours, subsequently extending into the neck and trunk. Physical exam was pertinent for blanching, erythematous macules, with well-demarcated borders on the affected areas, alongside scaling and skin denudation behind the right ear and upon the right upper chest. There was pain to light palpation in all affected areas. Rapid strep test results in the clinic were negative. The patient was sent home with instructions to initiate a clindamycin regimen and follow up after completion of treatment. Due to worsening rash the following day, the patient was taken to a tertiary care hospital, where she was evaluated and diagnosed with SSSS. This case demonstrates an occurrence of SSSS at an atypical age, underscoring the importance of including SSSS as a differential diagnosis for all pediatric rashes.

## Introduction

Staphylococcal scalded skin syndrome (SSSS), also referred to as Ritter’s disease, is an uncommon dermatological condition induced by toxin-producing strains of *Staphylococcus aureus* (*S. aureus*), affecting fewer than 1 in 1.8 million people [1-6]. This condition may be preceded by a prodrome, including fever, agitation, and fatigue [1-3]. Mucosal (conjunctival, nasal, pharyngeal) and sensitive (umbilical, genital, gluteal) areas serve as common sites for initial infection, spreading within 48 hours to the face and intertriginous regions, before soon affecting larger surfaces of the body [1,2]. Early epidermal changes typically involve erythematous blisters and bullae with a positive Nikolsky sign on examination [1,2,4,5]. Scaling and skin denudation may also be observed [1]. As the disease progresses, lesions may enter a second phase of desquamation, causing the skin to resemble scalded tissue, as suggested by the name [1]. With proper management, recovery occurs without scarring within two weeks [1-3]. Early diagnosis and treatment are imperative to prevent life-threatening complications such as dehydration or sepsis [1,4-5]. However, diagnosing SSSS may be difficult since pediatric skin concerns are common [4], and there exists a wide range of differential diagnoses to consider, including Stevens-Johnson syndrome, toxic epidermal necrolysis, bullous impetigo, allergic contact dermatitis, and scarlet fever, among others [1,2,5]. For prompt detection, another factor to consider involves the patient’s age, as SSSS is most prevalent in children under the age of two, with significantly less occurrence in children over the age of six [1-4,6]. This case demonstrates the occurrence of SSSS at an atypical age, underscoring the importance of including SSSS as a differential diagnosis for all pediatric rashes.

## Case presentation

**Figure 1:**
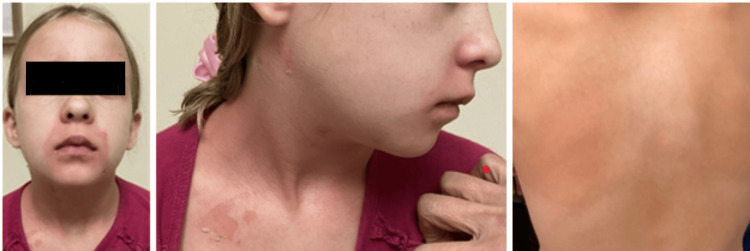
Initial encounter Well-demarcated, erythematous macules on the nose, chin, and forehead (left), scaling with denuded skin upon the right upper chest and post-auricular region (center), and blanching erythematous macules on the dorsal trunk (right). Consent for the publication of this image was received from the patient's guardian.

An unimmunized, afebrile, eight-year-old Caucasian female presented to the pediatric clinic with her mother to address concerns regarding a painful rash. This non-pruritic rash was reported to have begun on the face prior to spreading to the neck and trunk within 24 hours. At the time of presentation, the areas of rash were tender to light palpation, and the patient reported pain on donning her clothes earlier that morning. Physical exam was pertinent for an anxious and ill-appearing child with well-demarcated erythematous macules on the nose, chin, and forehead, blanching erythematous macules on the trunk, and scaling with denuded skin behind the right ear and upon the right upper chest. Erythema was also noted in the bilateral axillae, and two areas of skin, on the neck and post-auricular region, were open and draining (Figure 1). Otherwise, the patient had moist mucus membranes, a non-erythematous pharynx clear of exudate, and no lymphadenopathy of the neck. The exam of the ears, eyes, lungs, and heart was also non-contributory. The patient was not known to have any allergies to food or medication, and did not have pertinent past medical, surgical, family, or social history.

Rapid strep test results in the clinic were negative. Beta Strep Group A culture was also ordered with sampling from the pharynx; this culture returned negative. Additionally, a complete blood count (CBC), comprehensive metabolic panel (CMP), sedimentation rate, C-reactive protein (CRP), and Epstein-Barr virus (EBV) titer were ordered. At the parents’ request, a Lyme titer was also ordered. CBC returned within normal limits (WNL), bar an elevated absolute neutrophil count and reduced absolute lymphocyte count (Table 1). CMP returned WNL, bar an elevated creatinine level and elevated albumin/globulin (AG) ratio (Table 1). Sedimentation rate and CRP returned WNL, and the patient was negative for EBV and Lyme disease (Table 2). At the conclusion of the initial encounter, the leading differential diagnosis was scarlet fever, and the patient was started on a regimen of 75 mg/mL Clindamycin palmitate hydrochloric acid (HCL) solution reconstituted, which was taken 7 mL orally every 8 hours for 10 consecutive days. Patient follow-up was recommended after completion of the course or if any concerns were to arise beforehand.

**Table 1:** Complete blood count and chemistry panel values retrieved after initial primary care encounter, with reference ranges provided by the local hospital’s clinical laboratory BUN, blood urea nitrogen; A/G ratio, albumin/globulin ratio; AST, aspartate aminotransferase; ALT, alanine aminotransferase

	Patient’s Values	Reference Ranges
White Blood Cells	10.3 K/uL	3.7 – 10.5 K/uL
Red Blood Cells	4.72 M/uL	3.91 – 5.45 M/uL
Hemoglobin	13.6 g/dL	11.7 – 15.7 g/dL
Hematocrit	40.5 %	34.8 – 45.8 %
Platelet	311 K/uL	150 – 450 K/uL
Neutrophils (Absolute)	8.0 K/uL	1.2 – 6.0 K/uL
Lymphocytes (Absolute)	1.2 K/uL	1.3 – 3.7 K/uL
Potassium	4.4 mmol/L	3.5 – 5.2 mmol/L
Sodium	139 mmol/L	134 – 144 mmol/L
Chloride	100 mmol/L	96 – 106 mmol/L
Glucose	84 mg/dL	70 – 99 mg/dL
BUN	11 mg/dL	5 – 18 mg/dL
CO2	20 mmol/L	19 – 27 mmol/L
Creatinine	0.76 mg/dL	0.37 – 0.62 mg/dL
Albumin	4.8 g/dL	4.2 – 5.0 g/dL
Globulin	2.1 g/dL	1.5 – 4.5 g/dL
A/G Ratio	2.3	1.2 – 2.2
Total Protein	6.9 g/dL	6.0 – 8.5 g/dL
Alkaline Phosphatase	220 mmol/L	150 – 409 IU/L
AST	30	0 – 60 IU/L
ALT	17	0 – 28 IU/L

**Table 2:** Various supplemental values retrieved after the initial primary care encounter, with the reference range provided by the local hospital’s clinical laboratory. EBV, Epstein-Barr virus; Ab, antibody; VCA, viral capsid antigen; IgM, immunoglobulin M; IgG, immunoglobulin G; CIA, chemiluminescent immunoassay

	Patient’s Values	Reference Ranges
Sedimentation Rate	9 mm/hr	0 – 32 mm/hr
C-Reactive Protein	2 mg/L	0 – 9 mg/L
EBV Ab VCA, IgM	< 36.0 U/mL	Negative < 36.0 U/mL
EBV Ab VCA, IgG	< 18.0 U/mL	Negative < 18.0 U/mL
EBV Nuclear Antigen Ab, IgG	< 18.0 U/mL	Negative < 18.0 U/mL
Lyme Total Antibody CIA	Negative	Negative

Due to worsening facial rash on Day 2 of the regimen, the patient’s parent took them to the emergency department (ED) of a tertiary care hospital, where the patient was evaluated for and diagnosed with Staphylococcal scalded skin syndrome (SSSS). The ED recommended admission for hydration, but her parents opted for ED observation instead. The patient was sent home the same day after demonstrating stable vitals and the ability to hydrate herself. The decision was made to continue the prescribed antibiotic regimen by her pediatrician and to follow up in the office.

**Figure 2:**
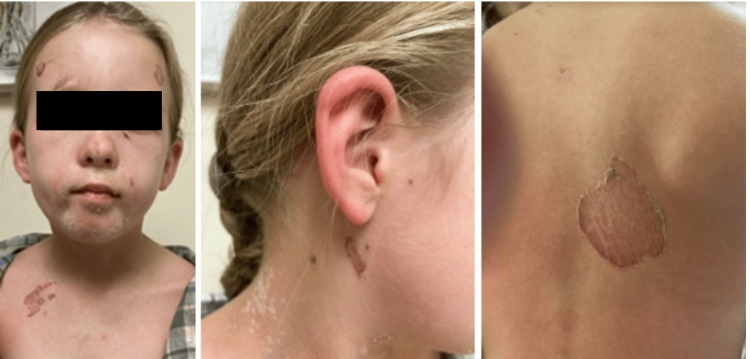
Three-day follow-up demonstrating changes on the nose, chin, forehead, right upper chest (left), post-auricular region (center), and dorsal trunk (right) Consent for the publication of this image was received from the patient's guardian.

On Day 4 of clindamycin usage, the patient and parent returned to the pediatric office for follow-up after an ED visit. During this visit, the patient was afebrile and in no acute distress. Physical exam was pertinent for dry, scaly lesions on the face, scattered erythematous macules on the face, neck, and trunk, and a brown blister-like lesion on the dorsal trunk (Figure 2). Lesions were not tender to palpation. With the established diagnosis of primary SSSS and improvement of skin condition, accompanied by a notable reduction in pain perception, the treatment plan was to continue the antibiotic regimen as initially prescribed. The patient and parent were advised to follow up as needed if the lesions were to worsen or if new concerns were to arise. There were no follow-up visits in the future regarding a recurrence or exacerbation of SSSS.

## Discussion

The pathogenesis of SSSS begins with an initial infection by *S. aureus *strains that produce exfoliative toxins A (ETA) and B (ETB) [1,2,4]. These toxins are serine proteases that disrupt skin integrity [1]. While *S. aureus* frequently colonizes mucosal and sensitive areas of the body, only about 5% of strains produce the toxins relevant to SSSS [1]. Once secreted at the infection site, ETA and ETB spread hematogenously to the stratum granulosum layer of the epidermis [1-4]. There, they accumulate and cleave desmoglein-1, a cadherin responsible for upholding adhesion between keratinocytes [1,2]. The resulting degradation of these adhesions leads to epidermolysis and the characteristic skin denudation and bullae seen in SSSS [1,2].

SSSS is primarily a clinical diagnosis, but a skin biopsy can be used to confirm the diagnosis if uncertainty exists [1,2,5]. For the patient in this case, initial in-clinic tests were negative, as expected for SSSS [1,2,4-5]. Despite being older than the typical age range, the patient exhibited classic symptoms, including the rapid progression of a painful rash across the face, neck, and trunk [1,4]. The patient’s lack of a significant medical history makes the exact factors contributing to her development of SSSS at age eight unclear. However, her unimmunized status sets her apart from most children in her age group, highlighting an area for further investigation.

Literature suggests that younger children, especially neonates, are more susceptible to SSSS due to underdeveloped immune systems that cannot generate antibodies against exfoliative toxins and limited renal capacity for toxin excretion [2,3]. By age eight, a patient with no previous renal concerns is likely to have sufficient renal function to clear toxins. However, the patient’s lack of immunizations may have contributed to a less robust immune response compared to her peers. Vaccinations save millions of lives annually by strengthening innate, humoral, and cell-mediated immunity [7]. Vaccines purportedly provide not only antigen-specific immune responses but also indirect benefits such as improving overall immune fitness [8]. This concept raises the question of whether vaccination may have broader effects on the immune system’s ability to respond to non-vaccine pathogens [8]. However, further research is required to establish whether there is a true correlation between vaccination status and immune protection against SSSS.

Regardless of the underlying cause, treatment protocols are consistent once the diagnosis of SSSS is established. In this case, the initial differential diagnosis was scarlet fever, prompting initiation of a clindamycin regimen. Following the diagnosis of SSSS, clindamycin was continued, yielding satisfactory results. Clindamycin inhibits bacterial protein synthesis, reducing toxin production, particularly those from *S. aureus* and *Streptococcus pyogenes* [1,9]. While effective in this case, recent literature suggests that SSSS-causing *S. aureus* strains may have higher resistance to clindamycin than other strains [1,6,9].

The standard first-line treatment for SSSS involves penicillinase-resistant penicillins such as nafcillin or oxacillin [1,2,4,5]. Second-line alternatives include first and second-generation cephalosporins [1,5]. For methicillin-resistant *S. aureus* (MRSA) or anaphylactic penicillin allergies, vancomycin or clindamycin may be utilized [1,2,5]. With early identification and treatment, the mortality rate for SSSS remains low at around 4% [1,2,4-5]. Hospitalization should be considered if complications such as dehydration or sepsis are suspected, ensuring prompt intervention to optimize patient outcomes [1,9].

## Conclusions

Increasing clinician awareness of the potential for Staphylococcal scalded skin syndrome (SSSS) across different pediatric age groups is crucial. While uncommon, this case provides evidence of its occurrence in a broader population than typically recognized. Given the single-case design, further series studies or registry analyses are needed to explore potential shifts in age distribution related to SSSS. Nonetheless, this case illustrates how early diagnosis and treatment, driven by heightened awareness, can improve outcomes and prevent potentially fatal complications.
